# Distant Ureteral Metastasis from Colon Adenocarcinoma: Report of a Case and Review of the Literature

**DOI:** 10.1155/2014/196425

**Published:** 2014-03-04

**Authors:** Ferakis Nikolaos, Anastasopoulos Panagiotis, Bouropoulos Konstantinos, Samaras Vassilios, Poulias Iraklis

**Affiliations:** ^1^Department of Urology, Korgialenio-Benakio Hellenic Red Cross Hospital, Athanasaki 1, 11526 Athens, Greece; ^2^Department of Pathology, Korgialenio-Benakio Hellenic Red Cross Hospital, Athanasaki 1, 11526 Athens, Greece

## Abstract

Carcinomas arising from organs neighbouring the ureter can directly infiltrate the ureter. Distant ureteral metastasis from colon adenocarcinoma is extremely rare and usually an incidental finding in performed autopsies. We report a case of a right ureteral metastasis in a 65-year-old Caucasian male with a history of rectal cancer for which he had been treated 4 years before. He presented with asymptomatic moderate right hydronephrosis. The patient underwent a right nephroureterectomy. Histology of the ureter revealed transmural adenocarcinoma with infiltration of the mucosa. Infiltration of the muscular coat of the bladder was found 2 years later. Thus, cystectomy and left ureterocutaneostomy were performed. The patient died 6 months later due to toxic megacolon during chemotherapy. The differential diagnosis of ureteral adenocarcinoma, especially in patients with previous history of colon adenocarcinoma, should include the possibility of distant metastasis from the primary colonic tumor.

## 1. Introduction

Distant metastases to the ureter may occur through lymphatic and/or blood vessels excluding ureteral involvement by direct extension [1–3]. These metastases have been reported more commonly arising from stomach, breast, lung, cervix, prostate, pancreas, and lymphoma [2–6]. Distant ureteral metastases from colon adenocarcinoma are considered to be very rare [2–10]. We present a case of this condition and review the English language literature.

## 2. Case Report

**Figure 1:**
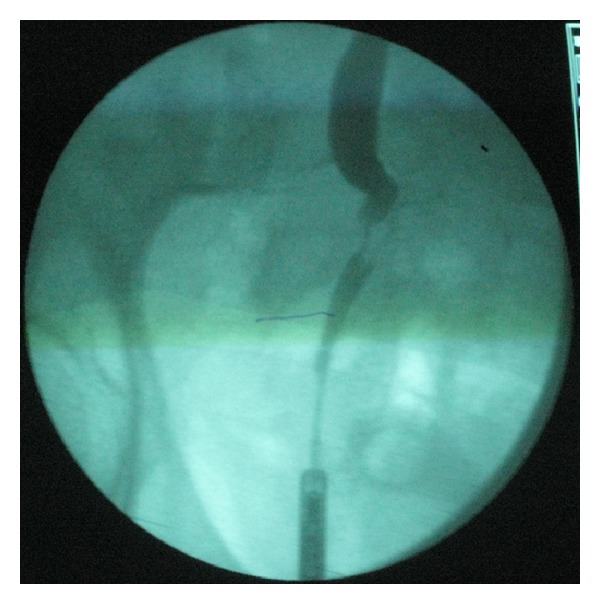
Ureteropyelogram revealing an obstruction of the lower right ureter, local ureteral dilatation, and intraluminal filling defect.

**Figure 2:**
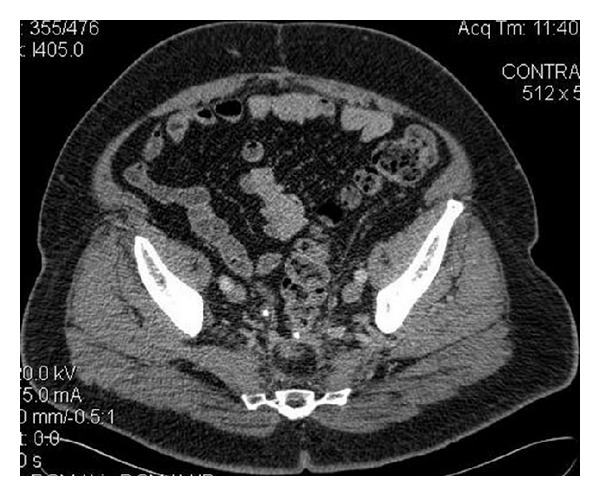
Computed tomography of the pelvis showing no evidence of extraureteral relapse.

In November 2005 a 61-year-old male patient underwent rectosigmoidectomy for nonmetastatic rectal cancer (grade II, stage T3N1MO), which was followed by chemotherapy (oxaliplatin and capecitabine) and radiotherapy. He had no signs of relapse during the follow-up. In May 2009 an asymptomatic moderate right hydronephrosis in follow-up computed tomography (CT) scan was revealed. The ureteropyelogram (Figure 1) showed a 1.5 cm filling defect in the lower right ureter. Ureteroscopy revealed a papillary lesion. Urine cytology showed malignant cells strongly suspicious for adenocarcinoma which correlated with the biopsy findings where immunohistochemistry demonstrated carcinoma with glandular differentiation and positivity for cytokeratin (CK) 20, fully compatible with a metastatic colonic type adenocarcinoma. The patient was fully evaluated with colonoscopy and CT (Figure 2) with no signs of local or distant relapse.

**Figure 3:**
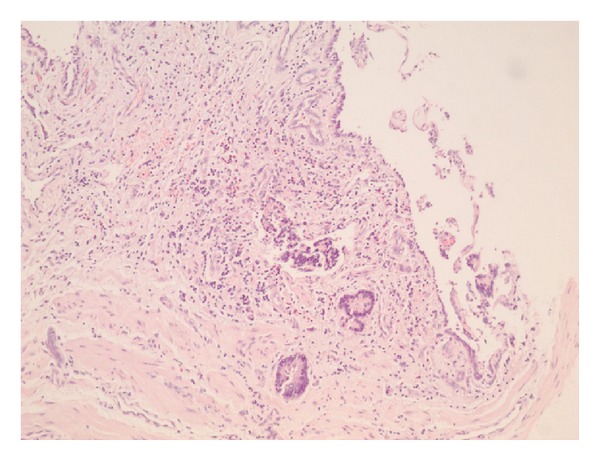
Neoplastic cellular population with an adenocarcinomatous pattern infiltrates the lamina propria of the ureter (hematoxylin/eosin ×10). Note the overlying atrophic or inflamed urothelium.

The patient underwent right nephroureterectomy. Histology revealed intraluminal adenocarcinoma (grade II) with infiltration of the mucosa, muscular layer, and adventitia (Figure 3). Periureteral tissue was free of tumor. This was followed by 6 cycles of chemotherapy. The patient was closely followed up afterwards until February 2012, where infiltration of the muscular coat of the bladder by adenocarcinoma (CK7−, CK20+), without other metastases, was found.

In March 2012 radical cystectomy and extended pelvic lymphadenectomy with left ureterocutaneostomy were performed. Foci of a moderately differentiated adenocarcinoma were identified in the bladder wall, localized both in the lamina propria as well as in the perivesical adipose tissue. Neoplastic vascular plugs and carcinomatous perineural infiltration were also found. The neoplastic population was immunohistochemically positive for CK7, CK20, carcinoembryonic antigen (CEA), *β*-catenin, and p53. The surgical margins were free of neoplastic disease. Severe lymphatic inflammatory reaction without evidence of lymph node metastasis was found (pT3apNO). The patient died 6 months later due to toxic megacolon during chemotherapy.

## 3. Discussion

Distant ureteral metastasis from primary colon adenocarcinoma was first reported in 1936 [2]. The most common sites of primary lesion are the breast, stomach, lung, cervix, prostate, pancreas, and lymphoma [2–6]. Reviewing the English language literature, reports describing true ureteral metastases from colon cancer are scarce [2–10]. Twenty-three cases have been published worldwide so far (Table 1).

In the first half of the last century, these ureteral metastases have been only described as incidental findings during autopsy due to the fact that the majority of patients were asymptomatic [2–6]. MacLean and Fowler reported an incidence of 2 cases in 10.223 performed autopsies [4]. Symptomatic patients most frequently described low back pain, renal colic, and/or anuria in cases of bilateral ureteral obstruction [5, 7, 8]. The fact that hematuria is a rather uncommon finding can be explained considering that ulceration of the ureteral mucosa is not likely to occur [2, 3, 6].

There are two interesting parameters that must be emphasized. First, in the majority of cases, there were synchronous metastatic lesions in other organs or lymph nodes (Table 1). In our case, a single ureteral metastasis with no evidence of other metastatic lesions was found 4 years after primary rectal adenocarcinoma and metachronous bladder adenocarcinoma developed 2 years later. Second, in our patient the ureteral lesion occupied not only the muscularis and the adventitia but the mucosa as well. According to the literature, mucosal involvement is extremely rare, with only 3 definite cases published so far [7, 9, 10].

Metastatic tumour cell deposition in the ureter has been described as infiltration of the periureteral soft tissue with compression to the ureteral wall, transmural infiltration, or, uncommonly, infiltration of the local mucosa [2, 3]. The precise mechanism responsible for distant ureteral metastasis and the factors promoting this process are still quite unknown. MacKenzie and Ratner were the first to propose as rigid criteria for the diagnosis of distant ureteral metastasis the finding of malignant cells in the perivascular lymphatic spaces or in the blood vessels around the ureter [1]. Later, Presman and Ehrlich modified the criterion as the demonstration of malignant cells in a portion of the ureteral wall together with the absence of any neoplasm in adjacent tissues [2]. Our patient showed no tumoral lesions in the periureteral and retroperitoneal space during surgery indicating that the disease was true distant ureteral metastasis from the colon according to the criterion of Presman and Ehrlich that has been widely accepted and used to date.

Metastatic ureteral adenocarcinoma after primary colon adenocarcinoma is aggressive and has a poor prognosis without evidence of long-term survival in the reported cases [2–10]. Due to the rarity of the disease there are no standardized treatment protocols but chemotherapy remains mainstay in combination with the surgical treatment.

In conclusion, the colon is a very rare site of origin for distant ureteral metastasis. Although this condition is infrequently encountered in clinical practice, the possibility of metastatic ureteral carcinoma must be considered in the differential diagnosis, once there is a clinical or radiographic evidence of ureteral obstruction in a patient, who has a history of colon cancer regardless the stage and previous therapy.

## Figures and Tables

**Table 1:** English literature published series regarding distant ureteral metastases after primary colon adenocarcinoma.

Author	Site of primary lesion (number of cases)	Age	Laterality	Symptom	Other sites of metastases	Segment of ureter involved	Ureteral infiltration	Time after diagnosis of colon cancer
Presman and Ehrlich [2]	Colon (2)	NA^†^	NA	NA	NA	NA	Periureteral and/or transmural	NA

Cohen et al. [3]	Colon (6) and rectum (1)	NA	NA	NA	Yes^‡^	NA	Periureteral and/or transmural	NA

MacLean and Fowler [4]	Rectum (2)	NA	NA	NA	NA	NA	Transmural	NA

Fitch et al. [5]	Rectum (1)	51	Right	Abdominal pain	No	Middle 1/3	Transmural	2 years

Richie et al. [6]	Colorectal (7)^‡^	NA	NA	NA	NA	NA	NA	NA

Williams and Chaffey [7]	Sigmoid (1)	69	Bilateral	Anuria-uraemia	Peritoneal, liver and lung nodules, omentum, and bowel implants	Right lower 1/3 Left middle 1/3	Transmural-mucosal	4 years

Brotherus and Westerlund [8]	Rectum (1)	70	Bilateral	Anuria	Skin metastasis around the colostomy	Right lower 1/3 Left lower 1/3	Transmural	2 years

Fazeli-Matin et al. [9]	Rectum (1)	57	Left	Sepsis	Mediastinal lymph nodes	Middle 1/3	Transmural-mucosal	3 years

Dickson et al. [10]	Colon (1)	78	Left	No	No	NA	Transmural-mucosal	4 years

^†^Not Available.

^‡^Not Specified.
